# Effects of Circuit Resistance Training Intensity on the Plasma Ghrelin to Obestatin Ratios in Healthy Young Women

**DOI:** 10.5812/ijem.2459

**Published:** 2012-04-20

**Authors:** Mehdi Hedayati, Marziyeh Saghebjoo, Abbass Ghanbari-Niaki

**Affiliations:** 1 Obesity Research Center, Research Institute for Endocrine Sciences, Shahid Beheshti University of Medical Sciences, Tehran, IR Iran; 2 Faculty of Physical Education and Sports Sciences, University of Birjand, Birjand, IR Iran; 3 Faculty of Physical Education and Sports Sciences, Mazandaran University, Mazandaran, IR Iran

**Keywords:** Obestatin, Ghrelin, Plasma, Women

## Abstract

**Background:**

Ghrelin and obestatin are orexigenic and anorexigenic peptides, respectively. It appears that an accurate balance between theses peptides is important for regulating energy homeostasis and body weight.

**Objectives:**

The aim of this study was to identify the possible mechanisms by which circuit resistance training influences energy homeostasis and weight control.

**Patients and Methods:**

Twenty-seven female students with the mean age of 22 ± 1.54 years and mean body mass index (BMI) of 20.76 ± 1.86 kg/m^2^ were selected and randomly divided into experimental and control groups. Subjects performed circuit resistance training with 40% and 80% of 1 repetition maximum (1RM) for 4 weeks. Total plasma ghrelin, obestatin, and glucose levels and the ghrelin to obestatin ratio were measured for all subjects before and after training.

**Results:**

One-way ANOVA tests showed that, the plasma ghrelin to obestatin ratio increased significantly in the 80% 1RM group (P < 0.05). Furthermore, a significant reduction of the plasma obestatin level was found in this group (P < 0.05).

**Conclusions:**

It appears that an energy deficit caused by circuit resistance training in 80% of the 1RM group resulted in the ghrelin precursor being increasingly used for ghrelin production. Thus, obestatin secretion decreased and the ghrelin to obestatin ratio increased in order to stimulate food intake and lost energy resource consumption to eventually restore the energy balance in the body.

## 1. Background

Energy balance is maintained through a complex network that includes central and peripheral factors. The ghrelin and obestatin peptides are 2 well-known peripheral factors that appear to play an important role in food intake and body weight regulation. Ghrelin is a 28 amino acid peptide that is mainly secreted by the gastric fundus cells into the blood (1, 2). Ghrelin affects hypothalamic satiety and hunger centers and stimulates food intake and weight gain. Research findings show that expression level of the ghrelin gene increases in the stomach during fasting, whereas release of the peptide decreases under satiety condition. In fact, plasma ghrelin levels decrease under positive energy balance conditions and increase under negative energy balance conditions (3-6). Recently, Zhang et al. (2005) identified a 23 amino acid peptide called obestatin (7). This peptide is encoded by the ghrelin gene through a change after ghrelin mRNA translation. Research shows that treatment of rodents with obestatin led to a negative energy balance by reducing food intake and gastric emptying. As a result, some investigators concluded that the conflicting effects of ghrelin and obestatin on weight and the undesirable impact of obestatin might be involved in the pathophysiology of obesity (7-10). In hormonal and metabolic studies, many questions still remain with regard to the changes in ghrelin and obestatin levels caused by exercise, since it is one of the factors influencing energy balance. Some studies have shown that exercise-induced weight loss and the subsequent loss of body mass index (BMI) can alter plasma ghrelin levels (4, 11, 12). Ghanbari-Niaki et al. study showed that ATP and the glycogen fraction from the liver of mice injected with ethionine increased plasma ghrelin concentrations, which can be considered as an important initiator of food intake (13). Several recent studies showed that an increased ghrelin to obestatin ratio has an important role in regulating energy balance, weight control, and the pathophysiology of obesity (6, 14-16). Altogether, the role of obestatin in body weight regulation and in the mechanisms underlying obesity is still unclear, the balance between ghrelin and obestatin plays an essential role in obesity and metabolic diseases (6). Guo et al. (2007) found that ghrelin and obestatin levels were lower but the ghrelin to obestatin ratio was higher in obese subjects than in normal-weight control groups. Additionally, recent findings by Zizzani et al. (2007) have demonstrated that, whereas fasting resulted in elevated ghrelin levels, it reduced obestatin levels, suggesting opposite effects on energy metabolism (17). The benefits of aerobic exercise training in reducing obesity have been well documented for decades, and aerobic training has been used as an exercise intervention to investigate the effects of training on peptides involved in energy balance (11-13). However, the resistance exercise training (RET) is a main part of the program for weight control and health, and it can simultaneously increase muscle strength (18, 19). However, the effects of training on peptides involved in energy balance are not known. It seems that the relationship between changes in the levels of these peptides and RET can provide an approach to achieve weight control.

## 2. Objectives

The aim of this study was to determine the effect of circuit RET with 40% and 80% 1RM (maximum weights that a muscle or a group of muscles can lift only once) on the ghrelin and obestatin levels and the plasma ghrelin to obestatin ratio in young women.

## 3. Patients and Methods

A quasi-experimental method was used for this study. The research design included pre-test and post-test in 2experimental and control groups. The study population comprised female physical education student volunteers. Among these, 30 individuals were selected voluntarily and purposefully. They were randomly divided into 2 experimental (N = 20) and control (N = 10) groups (during the study, 3 subjects were excluded because of their unwillingness to participate). The inclusion criteria for participants were lack of cardiovascular, respiratory, renal, and metabolic diseases. In addition, the subjects were not using steroid drugs or special diets (low calorie, low fat, or high protein diets). Since estrogen can influence ghrelin levels (20), all subjects had regular menstrual cycles and were similar to each other. In addition, until the beginning of this study, participants did not have a history of regular exercise with weights. Consent forms were obtained from all subjects. The subjects’ height and weight were measured and BMI was calculated by a formula. The percentage of body fat was determined with a caliper and the Jackson/Pollock three-point method and by measuring the subcutaneous fat at the triceps, abdomen, and suprailiac sites (Table 1).

**Table 1:** Individual Characteristics of Subjects in Experimental and Control Groups a

	Time	Control Group (n = 8)	40% 1RM b (n = 9)	80% 1RM b (n = 10)
Age, y	Pre-test	20.75 ± 1.04	23.22 ± 0.97	21.90 ± 1.52
Height, cm	Pre-test	161.75 ± 3.40	163.22 ± 6.18	162.6 ± 4.5
Weight, kg	Pre-test	52.6 ± 3.2	56.6 ± 6.7	55.3 ± 4.5
	Post-test	52.9 ± 2.1	57.3 ± 6.6	55.1 ± 5.6
BMI, kg/m^2^	Pre-test	20.1 ± 1.5	21.2 ± 2.3	20.8 ± 1.6
	Post-test	20.3 ± 1.3	21.5 ± 2.2	20.6 ± 2.3
Percentage of body fat	Pre-test	21.1 ± 1.6	21.2 ± 2.4	20.6 ± 2.3
	Post-test	21.3 ± 1.3	20.4 ± 2	20.6 ± 2.3

^a^Numbers are defined in terms of Mean ± SD

^b^Abbreviations: RM, Repetition maximum

Finally, 1RM values for the 9 movements in the experimental groups were determined using the following formula (21).

**Figure:**
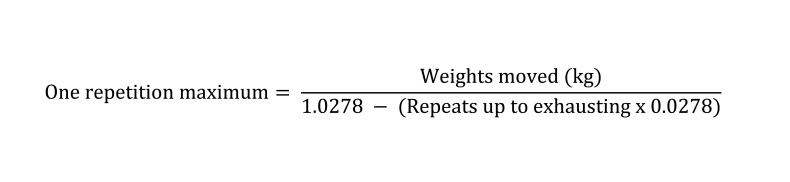


### 3.1 Exercise Training Protocol

The experimental group performed exercises in sessions starting at 8 AM sessions, 4 times a week, for 4 weeks, with 2 intensities-40% and 80%-of 1RM. The training program was designed using free weights and machines. These movements included chest press, leg press, seated rowing, overhead press, knee extension, triceps extension, leg curl, arm curl, and heel raise. Each training session included 3 circles, and in each circle, the above-mentioned 9 movements were performed consecutively. Each movement lasted for 30 s (with 8–11 repeats). The rest periods between 2 movements and 2 circles were 30 and 120 s, respectively. Each training session lasted for 50–55 min, including light warm-up without resistance working for 20 min, a weight-training program for 30 min, and a cool-down period of 5 min. Blood sampling was performed 24 h before the first training session and 48 h after the last training session in all 3 groups. Because of the effect of food type on the plasma ghrelin levels, fed subjects (who had dinner and breakfast) were considered identical before sampling (in terms of timing and the type of food intake). Breakfast (with about 500 kilocalories) was served 3–4 h before sampling, and subjects subsequently fasted until sampling. It should be mentioned that subjects were at the middle of the luteal phase (20–23 days after the onset of the menstrual cycle), which was determined on the basis of their menstrual cycles in the most recent 6 months after they were referred to the laboratory. Some studies have shown that estrogen affects plasma ghrelin levels (20). Considering that estrogen levels are less volatile in the middle of the luteal phase of a normal menstrual cycle, in order to prevent the interaction between estrogen and ghrelin levels, this stage was considered as the sampling time before and after training. Blood samples (10 ml) were obtained via the brachial vein (at 8 AM). Samples were collected in tubes containing anticoagulant (EDTA) and were immediately centrifuged (2000 rpm for 10 min). Plasma was used to measure ghrelin, obestatin, and glucose levels. Total plasma ghrelin levels were measured by sandwich ELISA, using human kits (USCN LIFE Science & Technology Company, Missouri, USA). The sensitivity of this method was 15.6 pg/mL. The intra-assay coefficient of variation was 7.4%. Obestatin plasma levels were measured by sandwich ELISA using U.S. Company (USCN LIFE Science & Technology Company, Missouri, USA) human kits. The sensitivity of this method was 78 pg/mL. The intra-assay coefficient of variation was 6.9%. Plasma glucose was measured using enzymatic colorimetry (glucose oxidase), test kits, and SELECTRA2 Pars devices. The sensitivity of this method was 1 mg/dL, and the intra-assay coefficient of variation was 1.2%. In the present study, Tecan Sunrise ELISA Reader (Austrian company) was used. Descriptive statistics were performed for data analysis. To use the appropriate statistical test, considering the sample size of the 3 groups, we first carried out the normal distribution/homogeneity of variance of the variables by using the histogram of the sample and also the Shapiro-Wilk test, which showed that the variables had a normal distribution. To compare the mean variations of variables in the experimental group before and after training, the parametric one-way analysis of variance (ANOVA) and the LSD post hoc test were used. Statistical calculations were carried out using the SPSS version 15. Significance level was set as P < 0.05.

## 4. Results

The Kolmogorov–Smirnov test was used to determine the normality of the distribution, and variables were found to be normally distributed. The ANOVA test was used to ensure that no significant differences existed among variables. The results obtained for ghrelin (F = 0.86, P = 0.44), obestatin (F = 1.87, P = 0.18), and the ghrelin to obestatin ratio (F = 2.78, P = 0.08) showed no significant correlation between the levels of the variables in the 3 groups at the pre-test phase. The changes in the plasma levels of total ghrelin, obestatin, the ghrelin to obestatin ratio, and glucose in subjects from the experimental and control groups are given in Table 2. A significant correlation exists between the plasma obestatin levels and the ghrelin to obestatin ratio in the participants (P < 0.05 and P < 0.012, respectively). The LSD post hoc test results showed that, compared to the control group, the experimental group with 80% intensity of 1RM showed a significant decrease in ghrelin levels and ghrelin to obestatin ratio and the experimental group with 40% intensity of 1RM showed a significant increase in these values. In addition, no significant differences were seen between the levels of total ghrelin and plasma glucose in the experimental and control groups in the pre-test and post-test phases (P = 0.88 and P = 0.1, respectively).

**Table 2:** Plasma Levels of Total Ghrelin, Obestatin, Ghrelin to Obestatin Ratio, and Glucose in Subjects

	Time	Control Group	40% 1RM d	80% 1RM d	P value a
Total ghrelin level, pg/mL	Pre-test	311 ± 162 b	414 ± 154	397 ± 195	0.88
	Post-test	316 ± 175	446 ± 186	451 ± 142	
Obestatin level, pg/mL	Pre-test	205 ± 48	110 ± 57	158 ± 143	0.05 c
	Post-test	229 ± 43	126 ± 64	86 ± 22	
Ghrelin to obestatin ratio	Pre-test	1.70 ± 1.30	4.06 ± 1.70	3.37 ± 1.83	0.012
	Post-test	1.30 ± 0.70	4.09 ± 1.25	5.66 ± 2.15	
Plasma glucose level, mg/100 mL	Pre-test	89.6 ± 8.7	87.2 ± 5.9	82.1 ± 5.0	0.11
	Post-test	84.5 ± 4.2	83.3 ± 3.5	83.1 ± 4.9	

^a^Considered significant

^b^Numbers are defined in terms of mean ± SD

^c^Significant difference between the groups

^d^Abbreviations: RM, Repetition maximum

## 5. Discussion

The findings of this study suggest that obestatin plasma levels and the ghrelin to obestatin ratio significantly decreased and increased, respectively, after 4 weeks of resistance exercise with 80% intensity of 1RM. In addition, ghrelin and plasma glucose levels did not change significantly after 4 weeks of resistance training. Several studies have shown that obestatin and ghrelin play important roles in regulating the energy balance and weight control (5, 6, 8). It seems that these 2 peptides have antagonistic actions with respect to food intake, weight gain, and adiposity. Many studies have demonstrated that ghrelin is sensitive to negative energy conditions and that it has an important role in balancing short- and long-term energy and glucose homeostasis (13). Since obesity is one of the biggest health concerns of modern societies, understanding the factors involved and the mechanisms by which it can be prevented can help promote public health and reduce medical expenses. Vicennati et al. (2007) showed that compared to normal-weight subjects, obese women have higher plasma obestatin levels and lower plasma ghrelin levels and ghrelin to obestatin ratio. They also found that the ghrelin to obestatin ratio is inversely correlated with BMI and the abdominal obesity distribution. Altogether, reduction of the ghrelin to obestatin ratio in obese women supports the hypothesis that ghrelin and obestatin imbalance may be related to the physiopathology of obesity (16). No clear evidence exists to explain the potential mechanism(s) by which the resistance training 1RM 80% affects the plasma ghrelin to obestatin ratios. However, some possible mechanisms that may be related to ghrelin may help understand our current findings. It is well established that ghrelin and obestatin are encoded by the preproghrelin gene and originate from the post-translational processing of the preproghrelin peptide. It is possible that a condition induced by exercise is able to disrupt the balance between ghrelin and obestatin production (22). In 2008, Mager et al. reported to have found increased expression of ghrelin mRNA and its receptors in lymphocytes after resistance exercise of intensities of 60% and 70% of 1RM (3 and 4 days a week, 1 and 2 times per session, 10–12 repeats at every turn) (22). It should be mentioned that some researchers have reported that human and rodent plasma obestatin levels are not affected by fasting or satiety (22, 23). Therefore, in the present study, changes in plasma obestatin levels do not appear to be related to a subject’s satiety or hunger. Different studies have shown that weight training leads to increased glycogen breakdown and energy deficit. Additionally, after heavy exercise, protein synthesis and glycogen reconstruction occur slowly (24). On the other hand, it has been reported that eccentric exercise leads to muscle damage and glycogen synthesis defects. Human and animal studies found a positive correlation between insulin increase and glucose transport by GLUT-4 in muscle cells. In fact, muscle GLUT-4 content was shown to be directly related to muscle glycogen levels; therefore, a defect in muscle glycogen re-synthesis after eccentric contractions might have occurred as a result of reduced GLUT-4 protein content, which was induced by muscle damage. The GLUT-4 protein content is one of the determinants of muscle glucose uptake (25-27). Therefore, we can say that exercise with 80% 1RM intensity leads to more muscle damage and thus reduces the muscle cell membrane GLUT-4 content. This can delay muscle glycogen re-synthesis, resulting in a negative energy balance in the body. Thus, it appears that conditions induced by high intensity resistance training result in the proghrelin processing being shifted toward more ghrelin production. As a result, obestatin secretion decreases and the ghrelin to obestatin ratio increases. This change can stimulate of food intake and lead to lost energy compensation, and eventually, the body’s energy balance is restored. Most studies have found that long-term exercise results in increased ghrelin plasma levels if weight loss is observed (4). In our study, we did not observe any significant increases in plasma ghrelin levels, and this can be explained by the fact that the participants did not lose weight during the training program. In fact, increased ghrelin levels probably act as a compensatory mechanism to restore the body weight to a set point (28). Some studies have reported that plasma levels of ghrelin increase almost twice during fasting, and fasting is one of the factors that creates a negative energy balance that can stimulate the expression of appetite peptides such as ghrelin (3, 29, 30). It seems that fasting at the time of sampling probably affected the alterations resulting from exercise. Therefore, in order to control the confounding effects of fasting, subjects received about 500 kilocalories 4 h before sampling. The overall findings of this study demonstrated that circuit resistance exercise with 80% intensity of 1RM leads to a significant reduction in plasma obestatin levels and increased ghrelin to obestatin ratio. These changes only occurred when exercise was performed at 80% intensity of 1RM. It seems that high-intensity resistance training results in a negative energy balance in response to energy shortages, because of muscle damage and a delay in reconstructing the muscle glycogen stores.
